# A Lecture on Hæmoptysis

**Published:** 1839-02-09

**Authors:** N. Chapman

**Affiliations:** Professor of the Theory and Practice of Physic, in the University of Pennsylvania


### MEDICAL EXAMINER.

DEVOTED TO MEDICINE, SURGERY, AND THE COLLATERAL SCIENCES.

No. 6.] PHILADELPHIA, SATURDAY, FEBRUARY 9, 1839. [Vol. II.

### A LECTURE ON HÆMOPTYSIS.

*By* N. Chapman, M. D., *Professor of the Theory and Practice of Physic, in the University of Pennsylvania.*

(Continued from page 70.)

Enough, perhaps, has been said in the preceding discussions, to convey my views of the pathology of hæmoptysis. It is essentially the same as that of hæmorrhage generally,—and as to its slight peculiarities, these need scarcely detain us. The mucous membrane of the lungs is the seat of much the larger proportion of cases,—and, it might be added, of the whole of genuine spontaneous hæmorrhage, with the exception of those of the cellular tissue of the pulmonary structure, which latter are so exceedingly rare, that I have seen only three, or, perhaps, four instances of it. Even such as proceed from tubercles, or other organic lesions of the lungs themselves, seem thus to be located—these lesions serving so to irritate the mucous membrane, as to cause the effusion.

In support of this doctrine, it were easy to extend the authorities on the subject, were it required. The language of Laennec, however, is so emphatic, that I cannot forbear to cite it. "Hæmoptysis," says he, "is now very generally considered as depending on some functional derangement of the bronchial membrane, which causes it to exhale blood, in place of its ordinary mucous secretion,"—and, speaking of the other form of it, he remarks, that "it is evidently an effusion of blood into the parenchyma of the lungs, or, in other words, into the air cells. From its exact resemblance to the effusion which takes place in cerebral apoplexy, I have thought the name, pulmonary apoplexy, very applicable to it.* Commonly, the effusion is into the cellular tissue, and may pass out through the bronchiæ communicating with the cells, though, occasionally, there is a rupture of the substance of the lung, and the blood escapes into the cavity of the pleura, of which Corvisart gives an example."

It is not, however, to be understood, that the doctrine I am sustaining, goes to the denial of hæmoptysis in other modes. That it may be induced by rupture from mechanical violence, or by an aneurism of the pulmonary arteries, or varicose state of the veins, is certain, and, perhaps, scarcely less so occasionally, when it attends the tubercular excavation, or ulceration of the lungs. But such cases seldom occur, and cannot be considered as spontaneous or vital hæmorrhage.

From the exhibition I have presented of the tendencies of hæmoptysis, it follows, that with the exception of the apoplectic species of it, which always requires the most prompt relief, it were of no great consequence to suppress it, unless the effusion be excessive, or occurs in debilitated states of the system. Generally, it will be found salutary, when moderate, producing very much the same effect as the artificial detraction of blood, and often supersedes the necessity for it. Determined, however, from any consideration to check the hæmorrhage, the indication, in the active form of it, is plainly to reduce vascular force, or remove local congestion, or phlogosis, for which purposes venesection appears peculiarly appropriate, and has the sanction of long and concurrent authority. Yet some have objected to it,—and, among others, the celebrated Heberden. Deliberately is it put by him as a question, to be solved by medical ingenuity, how the opening of a second vessel can check the flow from the one already ruptured? Not to advert particularly to the erroneous predication of this hæmorrhage being dependent on rhexis or rupture, I shall remark, that this is a sophism, unworthy of that candid physician. Whatever might be the difficulty of explanation, he well knew that the efficacy of bleeding had been amply demonstrated. Really, it seems to me, that the case involves no dark enigma. Distinct from other modes in which it operates, such as the reduction of the amount of blood, and the force of the circulation, by opening a vein in another part of the body, the flow of blood is invited to it, and thus, on the principle of revulsion, contributes largely to check the hæmorrhage. The solution of the problem, however, is immaterial. Bleeding is sufficiently admitted to be useful, and with this, we may, at present, be satisfied.

To meet the more violent attacks, it is indispensably necessary that the detraction of blood be large. Boerhaave says, "that hæmoptysis is cured by copious bleeding, every third day, for four times, or till the inflammatory crust disappears from the blood." The latter clause of the aphorism is sound, and conveys excellent practical advice,—though why this ternery recurrence to the remedy? More, however, to be condemned, are the small and repeated bleedings advised by some practitioners. They harrass, and weaken, without at all contributing to the cure. Did this require any enforcement, it might

*In passing on, I will merely remark, that this term is not original with Laennec, as he seems to suppose. It was, I know, employed forty years ago, by the late Professor Rush, to express those heavy congestions to which the lungs are liable, in contradistinction to peripneumony, an inflammation of the same texture, and in this sense, has ever since been retained among us. Laennec, however, I believe, was the first to apply it to pulmonary hæmorrhage, he having, too, the much higher credit of the original detection of this particular lesion.

be had in the history of numerous cases on record, where such practice was pursued ineffectually for a length of time, among which I have read an account of one, in a late number of an English journal, where three hundred and fifty-seven ounces of blood were drawn away in seventeen days. Now, a large bleeding would probably, at once, have proved decisive, and all this time and exhaustion spared. It is my practice, when called to a bad case of hæmoptysis, in which I think it expedient promptly to effect relief, to take away directly so much blood as to make a decided impression, or, in other words, to attain the end for which it is designed. Nothing short of this will be effectual in such hæmorrhages.

By some of the authorities, it is recommended to restrict the use of the lancet to cases only that are marked by fulness and activity of the circulation, with vigour of constitution. But, though here more urgently required, were the practice encumbered by such a limitation, the consequence would be eminently pernicious. It so happens, indeed, that a large proportion of the cases of hæmorrhage, and of active hæmorrhage, too, is attended not so much by redundancy of blood, as an unequal distribution of it, and this in individuals very far from being robust. Driven into the lungs in this undue quantity, which they are unable at once to return, and still more pushed on by the *vis à tergo*, which this congestion increases, the blood must force itself out, and continue to flow while such a condition of things endures. To remove this topical accumulation, as well as to restore, an equilibrium in the circulation, venesection is the appropriate remedy. We see it most strikingly evinced in cerebral apoplexy, and in many other analogous cases.

Nor, in another view, is the loss of blood less important. The lungs, in active hæmoptysis, are inflamed, or highly disposed to take on inflammation. Cases are exceedingly familiar, which, in the commencement, exciting little solicitude, have, from a neglect of this sort of depletion, run on to the establishment of inveterate and even fatal lesions. Whether with a view, therefore, to immediate relief from the pressing evil, or as a measure of prevention against more serious mischiefs, depletion in the way, and to the extent I have advised, is imperatively demanded.

As a substitute for the lancet in less urgent cases, or on considerable reduction by it of vascular force, leeches or cups to the chest may be usefully employed.

Of the late writers, some seem to prefer the detraction of blood from the remote parts, as the vulva or anus, on the principle of derivation. Excepting, however, in those instances occasioned by a suppression of the catamenia or hæmorrhois, (and here, probably, answering better, it should be adopted,) I think an application over the seat of the affection, is more certain and advantageous. But it is maintained, and by very high authority, that both topical and general bleeding, instead of proving remedial, serve, on the contrary, sometimes, to excite hæmorrhage. "I have noticed," says Laennec, "a return of the menses, and an increase of the menorrhagia, during an applicatian of leeches to the epigastrium. General bleedings, more particularly those of small extent, appear, occasionally, to have a similar effect on hæmoptysis." Clarke, one of the most respectable of the late writers, also states, that in a plethoric person, threatened with apoplexy of the brain, or pulmonary hæmorrhage, the use of leeches may, and he believes, frequently does decide the occurrence of the very disease it was intended to prevent, in proof of which, cases are given by him. Creditably as the position is sustained, I still doubt exceedingly the accuracy of the fact, and am disposed to consider such occurrences as rather coincidences, than effects. They are, at least, contradictory to the tenor of observation and experience. But while thus vindicating the utility, and even absolute necessity of bleeding, I am no less sensible that there are limits, beyond which it ought not to be carried. As in profuse or repeated hæmorrhage itself, any great excess in the operation is followed by the febrile movement, unequal distribution in the circulation, with a tendency to further effusions, or even, sometimes, to a general vitiation or cachexy of system. Counter-irritation, by a vesicatory to the chest, will supersede the necessity of any undue loss of blood, and in every view, is so important, that it should not be neglected.

Cold applications to the thorax, and particularly under the arm-pits, have been proposed as further means. No part is more susceptible than the axilla, and such applications are said to prove very effectual. The origin of the practice may be traced to an Italian writer early in the last century, who directs the naked breast to be covered with sponges dipped in cold water. It has indeed been recommended, on very urgent occasions, to wrap the whole body in a sheet wet with cold vinegar or water, or even to resort to immersion. The late Dr. Thomas Bond, of this city, a distinguished, though an eccentric, physician, is said to have pursued this course successfully. Bennet, on the contrary, tells us, that the cold bath is perilous. Two objections have been raised against the use of cold in any way, that it is calculated to repel the blood from the periphery to the centre, and thereby aggravate the hæmorrhage, and, subsequently, to induce catarrh or pneumonic inflammation, the lungs, at all times, being exceedingly intolerant of cold, in whatever manner applied, and the more so, in a predisposed state to disease. Nevertheless, such an impression on the skin, may, through the medium of sympathy, possibly constringe or otherwise close the bleeding vessels, and thus operate beneficially. An effect of this kind is conspicuously displayed in uterine floodings, and it should also be recollected, that cold applications to the surface are unquestionably, sometimes, of the greatest service, in the phlogosis of the contents of the other cavities of the body, which, on the same principle of driving the blood inwardly, would, were it not for the lessons of experience, be equally contraindicated. The cold bath, however, I should still very reluctantly try, except in the extremest emergencies, and where other measures had utterly failed.

Greater authority have we for the use of cold drinks, which seem to have been employed from the earliest down to the present times. No one, perhaps, has borne such strong testimony in their behalf as Martin Ghisi, he who first described croup. Cases of the most profuse hæmorrhage are reported by him, which were very speedily checked by a repetition of a cup of iced water every fifteen minutes. My own experience confirms this statement to the fullest extent.

It was an early, and for a very long time, an established practice, to apply ligatures to the legs, thighs, and arms, promptly to suppress the hæmorrhage. "By this means," says Van Swieten, "a considerable part of the blood is retained in the limbs, and a less quantity returns to the heart." Erasistratus probably introduced the remedy which has been commended by Moreton, Lieutaud, Bursenius, &c.* Being abandoned, I presume, it was ultimately found not to answer the end, or came to be supplanted by others of superior efficacy. Of these, among the more prominent, is the muriate of soda, a tea-spoonful or more, every five, ten, or fifteen minutes in substance. Dissolved in the mouth, and gradually swallowed, it is supposed to create a stronger impression on the parts with which the vessels of the lungs have the most intimate sympathy.

Few articles, perhaps, are more popular in hæmoptysis than the nitrate of potash. It is one of the most common of the domestic remedies in this city, and there is abundance of professional authority among the foreign writers in favour of it. From its general properties, we should presume that it has no direct control over a flow of blood. The quality of astringency it certainly does not possess. Yet it is often prescribed under such a conviction, and in a large quantity. As much as an ounce of it has been given in the day. Thus, freely exhibited, its utility being confirmed, the explanation of its modus operandi must be sought, I think, in the irritation caused in the stomach, by which action is diverted from the pulmonary vessels.

Even better established, perhaps, is the reputation of the acetate of lead. The mode of giving it, is in the dose of a grain or two, with a small portion of opium, at short intervals. Conformably to our general notions, a large dose of the article ought to accomplish much more, and certainly it might be taken with safety. Yet on one occasion, I directed twenty grains of it without any good effect. As in the case of mercury, may not the action of the lead be influenced by the quantity? The former is a salivant or purgative, according to the dose—and the latter may prove astringent or otherwise, in the same way.

* Vid. Celsus.

More particularly, I am inclined to think so, from having observed, in several instances, where very large amounts of it had been taken through mistake, that it operated altogether as a purge.

To the utility of one of the preparations of zinc, namely, the vitriolic solution, a compound of the sulphate of zinc and the sulphate of alumine, in this hæmorrhage, we have the evidence of Mosely, and of the late Professor Barton, strongly and unreservedly expressed. Of my own knowledge, I can say nothing in its favour, and on the same footing would I place the preparations of copper, so highly praised at one period.

By some practitioners, the efficacy of another class of remedies, the narcotics, is fully accredited. No benefit I presume, would result from the henbane and hemlock, though commended. But opiates promise more. It has been alleged against them, I am aware, that they are stimulant, and hence improper in this case. But, while granting this property to them, let it be recollected, that they have the effect of assuaging pain, and doing away irritation, by which excitement is so tempered, that they may do good, where, influenced by general principles, they would be prohibited. Nevertheless, I am not prepared by my narrow experience, to vindicate to the full extent, the propriety of this practice. in hæmoptysis. Whenever I have directed it in the early stage of the disease, there has been great irritation of the pulmonary organs, attended with spasmodic cough. No one will dispute its propriety under these circumstances; and here I would, at present, let it rest.

Digitalis, from its influence over the circulation, has been extolled in active hæmorrhages of every description. But as a substitute for the lancet, for which it is proposed, I know it is totally ineffectual, and ought never to be trusted. Even where vascular action is reduced by direct depletion, it has appeared to me very precarious, and decidedly inferior to many other remedies. In the ordinary dose, much time elapses before the pulse feels its influence, and if it be increased largely, there is such general prostration, with relaxation of the vessels, that a more copious effusion may be apprehended. An egregious error has been committed in the various applications of this medicine to hæmoptysis. The case to which alone it is suited, will hereafter be indicated.

Emetics have been employed in hæmoptysis with great success. My experience in regard to them is, however, chiefly confined to less active hæmorrhage, to which I believe them singularly well adapted, and what I have further to say in regard to them, I shall postpone, until I reach that part of the subject.

Doubting, as the generality of practitioners do, the propriety of emetics in active hæmoptysis, there is almost an undivided opinion as to their utility in small doses. The whole of this set of medicines may, perhaps, be applicable. Tartarized antimony is much employed, particularly in highly febrile states.*

Generally speaking, however, ipecacuanha is preferrable to any of the antimonials. Distinct from the power which it in common possesses with these preparations, of depressing vascular action, it seems also, to exercise a positive control over the hæmorrhagic disposition. Combined with the acetate of lead and opium, it sometimes proves more efficient than either article separately.

Nothing has hitherto been said of purging, which, however, whether we have regard to the reduction of vascular force, or the removal of local congestion, or the irritation induced by constipation, is not to be omitted. By Sydenham, who urged it to a considerable extent, and with the very active articles, its value is strongly insisted on. Laennec also affirms, that a drastic cathartic or enema, frequently checks the hæmorrhagic *molimen*, especially if productive of faintness, and altogether highly commends the practice. Castor oil, however, if the stomach be in a state retentive of it, answers well. Magnesia, which is sometimes selected, is apt, on account of its huskiness, by tickling the fauces, to excite coughing, should be avoided, or very carefully comminuted and mixed in the preparation.

The remedies mentioned, or some of them, at least, are those of the highest repute, with the design chiefly of suppressing the flow of blood. It is obvious, however, that on the accomplishment of this end, some further treatment may be demanded. The system, for the most part, is left febrile, or at least too highly excited, and to do away this state, an important consideration, a recurrence may be had to the mild febrifuge remedies, among which the antimonials are the best, alone or with the nitrate of potash. This is the occasion where I have principally used these articles, and to which I deem them exceedingly appropriate. Exciting diaphoresis or diuresis, especially, they hardly ever fail of producing the most unequivocal advantage. To appease the cough or pectoral irritation, opiates and demulcents may be required.

Meeting with considerable depravation of the primæ viæ, indicated by the furred tongue, morbid secretions, habitual constipation, &c., which is not uncommon, the proper treatment is by three or four grains of the blue pill at night, worked off the next morning by a laxative.

(To be continued.)

* The exorbitant use of this article, after the mode of Razori, was tried by Laennec, who reports, that though it appeared to lessen the discharge, it did not produce the same admirable results as in the case of pneumonia, and some other purely inflammatory diseases.

